# Differential Effects of Pre-Stroke Antithrombotic Medication on Clinical Outcomes of Patients with Hyperhomocysteinemia and First-Ever Stroke Versus Recurrent Stroke

**DOI:** 10.3390/jcm14196984

**Published:** 2025-10-02

**Authors:** Jungmin So, Sang-Hun Lee, Jin-Man Jung, Moon-Ho Park

**Affiliations:** Department of Neurology, Korea University Ansan Hospital, Ansan 15355, Republic of Korea

**Keywords:** homocysteine, ischemic stroke, recurrence, prognosis, antithrombotic

## Abstract

**Background/Objectives**: The associations between plasma homocysteine and pre-stroke antithrombotic medication and the effects these have on clinical outcomes of patients undergoing ischemic stroke remains unclear. This study aimed to evaluate the combined effect of plasma homocysteine levels and the use of pre-stroke antithrombotic medication on the clinical outcomes of patients experiencing first-ever and recurrent ischemic strokes. **Methods**: Anonymized data from consecutive patients who experienced ischemic stroke and had their plasma homocysteine levels evaluated were retrospectively analyzed. Pre-stroke antithrombotic medication status, clinical variables potentially influencing homocysteine concentrations, and stroke recurrence data were collected. Clinical outcomes were assessed using the modified Rankin Scale 3 months after stroke onset. The association between hyperhomocysteinemia and clinical outcomes was evaluated using logistic regression models. **Results**: Hyperhomocysteinemia was significantly associated with unfavorable clinical outcomes (adjusted odds ratio [aOR], 1.32; 95% confidence interval, 1.04–1.69) in the 2767 patients who were analyzed. The absence of pre-stroke antithrombotic medication use was associated with unfavorable outcomes (aOR range, 1.29–1.56), specifically in patients with first-ever stroke (aOR range, 1.45–1.64) but not in patients with recurrent strokes (aOR range, 0.70–1.04). **Conclusions**: Hyperhomocysteinemia and non-use of pre-stroke antithrombotic medication were significantly related to unfavorable outcomes in patients experiencing their first-ever stroke. These findings might provide prognostic insights into stroke management and patient stratification.

## 1. Introduction

Stroke is the third leading cause of death and the fourth leading cause of disability worldwide [1]. Its global prevalence included approximately 93.8 million cases in 2021 [1], and its overall incidence in South Korea was 232 per 100,000 individuals in 2018 [2]. Despite ongoing efforts to improve stroke management, these patients are still at a substantial risk of unfavorable clinical outcomes.

Homocysteine is a sulfur-containing toxic intermediate of the methionine metabolic pathway; increased total plasma levels from this intermediate are associated with an increased risk of atherothrombotic cardiovascular and cerebrovascular diseases [3,4,5]. Elevated homocysteine levels, known as hyperhomocysteinemia, can promote atherogenesis and thrombogenesis through multiple pathological mechanisms, including changes in coagulation pathways, tissue factor expression, fibrinolysis, endothelial dysfunction, platelet activation, proinflammatory effects, and smooth vascular muscle proliferation [5,6,7]. A meta-analysis of observational studies has reported that these increased levels are related not only to an increased risk of stroke [8] but also to unfavorable clinical outcomes after experiencing stroke [9,10,11,12].

Several trials have investigated direct homocysteine-lowering therapies with folic acid, vitamin B_6_, and vitamin B_12_; however, they have demonstrated no benefits and revealed inconsistent results regarding the prevention of cardiovascular and cerebrovascular diseases [13,14,15,16,17,18]. In addition to direct homocysteine-lowering interventions, indirect interactions between homocysteine levels and antithrombotic medications are also related to cardiovascular risk [18,19,20,21,22,23]. However, limited data are available regarding the combined effect of homocysteine levels and antithrombotic medication use on stroke prognosis, particularly in terms of patient disability.

This study aimed to investigate whether pre-stroke antithrombotic medications used for primary and secondary prevention of stroke influence the clinical outcomes of patients with stroke and hyperhomocysteinemia.

## 2. Materials and Methods

### 2.1. Participants

In this retrospective study, patient data were obtained anonymously from the Stroke Center of Korea University Ansan Hospital [24]. According to the criteria from the World Health Organization [25] and the Korea Stroke Society [2], stroke is defined as rapidly developing clinical signs of focal or global cerebral dysfunction lasting at least 24 h or leading to death without any apparent non-vascular cause.

Ischemic stroke is diagnosed based on clinical features consistent with focal cerebral ischemia and supported by imaging evidence of corresponding lesions on diffusion-weighted magnetic resonance imaging (DWI) with related low signal intensity on the apparent diffusion coefficient. These findings are assessed together with a compatible clinical history and neurological findings. Similarly, recurrent stroke is characterized as the development of a new acute neurologic deficit of vascular origin or the significant worsening of a pre-existing deficit not attributable to a brain shift, edema, hemorrhagic transformation, concurrent illness, hypoxia, or drug toxicity. Such recurrence requires confirmation via neuroimaging that demonstrates recent infarction occurring at least 24 h after the onset of the index event [26]. Patients were included in this study if they experienced an ischemic stroke and had documented plasma homocysteine levels at the time of discharge between March 2014 and December 2021. They were excluded if they had a transient ischemic attack, intracranial hemorrhage, subarachnoid hemorrhage, or subdural hematoma; they were unable to undergo magnetic resonance imaging (MRI); or they refused to participate in diagnostic tests to determine stroke etiology, with incomplete laboratory evaluations.

All patients underwent standard systemic evaluations, including routine laboratory tests, chest radiography, 12-lead electrocardiography, and neuroimaging studies. Routine laboratory assessments included a complete blood count, electrolyte panel, glucose, renal and hepatic function tests, lipid profile, and homocysteine measurement. The blood samples for these laboratory tests were collected from all participants during the morning following admission, after a fasting period of at least 8 h. Additional evaluations, including transcranial Doppler ultrasound, carotid duplex sonography, transthoracic or transesophageal echocardiography, and 24 h Holter monitoring, were selectively performed according to clinical indications. Neuroimaging techniques included brain computed tomography (CT) and/or MRI. Each patient underwent at least one form of vascular imaging, such as conventional angiography, magnetic resonance angiography, CT angiography, or duplex ultrasound imaging.

The study protocol was approved and supervised by the Ethics Review Committee of Korea University Ansan Hospital (Approval number: 2025AS0133). This study was compliant with the principles of the Declaration of Helsinki. Given the retrospective design and the anonymized use of patient data, the requirement for informed consent was waived by the ethics committee.

### 2.2. Homocysteine Determination

Plasma homocysteine levels were routinely measured in all patients within 2–7 days after stroke onset in accordance with previously described methods [27]. Briefly, venous blood samples were drawn into tubes containing ethylenediaminetetraacetic acid after 8–10 h of overnight fasting and immediately centrifuged. Plasma was separated from cellular components, placed on ice, and stored at −4 °C until analysis. Plasma homocysteine concentrations were measured using an automated ADVIA Centaur immunoassay system (Bayer Healthcare LLC, Tarrytown, NY, USA) via a direct chemiluminescence method. All measurements were performed blindly for clinical diagnoses and other results.

### 2.3. Clinical Assessment

The demographic and clinical data collected at baseline were age, sex, hypertension, diabetes mellitus, dyslipidemia, atrial fibrillation, reduced kidney function, hyperhomocysteinemia, leukocytosis, anemia, stroke subtypes, antithrombotic medication use, initial severity of stroke, and recanalization therapy.

Age at admission was categorized as follows: <65 and ≥65 years. Hypertension is defined as systolic blood pressure of ≥140 mmHg, diastolic blood pressure of ≥90 mmHg, or a previous diagnosis or prescription of antihypertensive medication. Diabetes mellitus is defined as a fasting serum glucose level of ≥126 mg/dL, a non-fasting serum glucose level of ≥200 mg/dL, a glycated hemoglobin (HbA1c) level of ≥6.5%, or a documented history of insulin therapy and/or oral hypoglycemic medication. Dyslipidemia is defined as a total cholesterol level of ≥200 mg/dL. Atrial fibrillation is defined as a persistent atrial arrhythmia characterized by irregular R-R intervals without distinct repetitive P waves confirmed by electrocardiography, 24 h Holter monitoring, or continuous electrocardiography monitoring during hospitalization. Reduced kidney function is defined as an estimated glomerular filtration rate of less than 60 mL/min per 1.73 m^2^ calculated using the Chronic Kidney Disease Epidemiology Collaboration equation. Hyperhomocysteinemia is defined as a plasma total homocysteine concentration of ≥15 μmol/L. Leukocytosis is defined as a white blood cell count of >12,000/μL. Anemia is defined as hemoglobin levels of <12 g/dL in females and <13 g/dL in males.

Stroke subtypes were classified into five etiological categories according to the Trial of Org 10172 in Acute Stroke Treatment (TOAST) criteria [28]: large artery disease (LAD), cardioembolism (CE), small vessel occlusion (SVO), stroke of another determined etiology (OD), and stroke of undetermined etiology (UD). LAD is diagnosed when brain imaging demonstrates substantial stenosis (>50%) or occlusion of a major ipsilateral artery presumed to be attributed to atherosclerosis and accompanied by corresponding clinical symptoms and DWI lesions, but without a cardiogenic source of embolism. CE is defined as cerebral infarction attributable to medium- or high-risk cardiac sources of emboli, without significant ipsilateral arterial stenosis or occlusion. SVO is identified by the presence of classical lacunar clinical syndromes and a lesion diameter of <20 mm confined to the territory of perforating arteries in subcortical regions or the brainstem and without cortical dysfunction or evidence of LAD or CE. Patients were classified as OD if their strokes were the result of uncommon causes, such as vasculopathies or hematological and coagulation disorders, without other identified etiologies. UD was assigned when patients had multiple potential causes, incomplete evaluations, or negative evaluations (cryptogenic stroke). Subtype classifications were reviewed independently by at least two neurologists, and consensus discussions were held when required. The final stroke subtype classification was confirmed through monthly stroke registry meetings.

Data on the pre-stroke use of antithrombotic medications, including antiplatelet therapy (APT; aspirin, clopidogrel, cilostazol, triflusal, or combinations) and oral anticoagulants (OAC; warfarin, dabigatran, apixaban, rivaroxaban, or edoxaban), were collected. The severity of neurological deficits at admission was assessed using the National Institutes of Health Stroke Scale (NIHSS) [29], where an initial NIHSS score of ≥5 was classified as poor initial NIHSS. Recanalization therapy was categorized as intravenous thrombolysis or intra-arterial thrombectomy.

### 2.4. Clinical Outcomes

Clinical outcomes were assessed using the modified Rankin Scale (mRS): a seven-point scale ranging from 0 (no symptoms) to 5 (severe disability) and 6 (death) [30]. mRS scores were recorded at discharge (discharge mRS) and 3 months (90 days) after the onset of stroke (3-month mRS). Clinical outcomes were divided into favorable (3-month mRS score of ≤2 or equal to discharge mRS) and unfavorable (3-month mRS score between 3 and 6) categories.

### 2.5. Statistical Analysis

Data were presented as frequencies and percentages for categorical variables and as medians with interquartile ranges for continuous variables. For stroke subtype analysis, patients classified as having stroke of OD and UD were combined into one subgroup (OD + UD) to achieve subgroup sizes comparable with other categories and minimize heterogeneity. Differences in baseline characteristics between clinical outcome groups were examined using Pearson’s chi-square (*χ*^2^) test or Fisher’s exact test as appropriate. Pairwise subgroup comparisons were performed using a *z*-test with Bonferroni correction to adjust for multiple comparisons.

The factors associated with clinical outcomes were investigated using univariable and multivariable binomial logistic regression analyses, and crude and adjusted odds ratios (aORs) with corresponding 95% confidence intervals (CIs) were recorded. A multivariable logistic regression model incorporating backward elimination procedures was used to examine the effects of different antithrombotic medications and identify significant covariates. Variables with *p* < 0.1 in the initial univariable analyses were selected for inclusion in the multivariable model.

Propensity score matching (PSM) analysis was conducted to validate our findings after controlling for potential confounders. PSM was performed to balance the baseline characteristics between the groups with favorable and unfavorable outcomes. One-to-one nearest-neighbor matching without replacement was applied using a caliper width of 0.25 times the standard deviation of the propensity score’s logit. Covariate balance after matching was assessed using the standardized mean difference (SMD), where an absolute SMD value of <0.1 indicates an acceptable balance. Propensity scores were derived from a multivariable logistic regression model that incorporated the following covariates: age, sex, hypertension, diabetes mellitus, atrial fibrillation, reduced kidney function, leukocytosis, anemia, recurrent stroke, stroke subtypes, poor initial NIHSS, and recanalization therapy.

Statistical significance was defined as two-tailed *p* < 0.05. Data were statistically analyzed using SPSS version 20.0 (IBM SPSS, Chicago, IL, USA) and R software version 4.3.3 (R Foundation for Statistical Computing, Vienna, Austria).

## 3. Results

**Figure 1:**
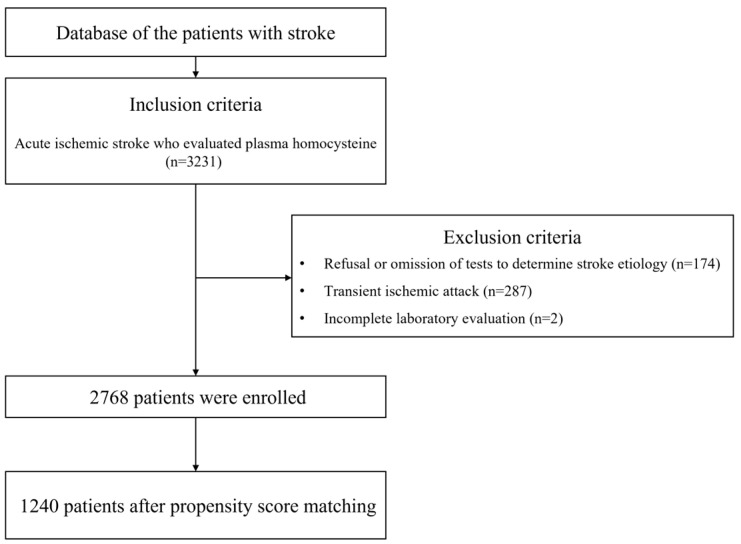
Flowchart of patient inclusion and exclusion.

During the study period, 3231 patients were enrolled, of which 463 were excluded based on prespecified criteria (Figure 1).

Consequently, 2768 patients were included in the final analysis, comprising 1739 (62.8%) males with a median age of 65.0 [56.0–77.0] years. Stroke subtypes included LAD in 608 (22.0%) patients, CE in 485 (17.5%) patients, SVO in 884 (31.9%) patients, and OD+UD in 791 (28.6%) patients. For pre-stroke antithrombotic medication, 413 (14.9%) patients used aspirin, 619 patients (22.4%) received any APT, and 103 patients (3.7%) used any OAC. Overall, 699 (25.3%) patients received either APT or OAC prior to the onset of stroke.

**Figure 2:**
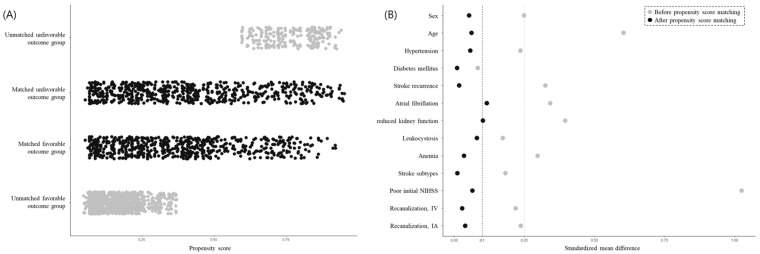
Distribution of propensity scores and balance measures after propensity score matching. Patients with stroke were classified into two groups of favorable outcomes (3-month mRS of ≤2 or equal to discharge mRS) and unfavorable outcomes (3-month mRS of ≥3). After propensity score matching was conducted, the favorable and unfavorable outcome groups comprised 620 patients who underwent stroke (**A**) and were matched such that the standardized mean differences between most background factors were within the cut-off value of 0.1 (**B**). Abbreviations: NIHSS, National Institutes of Health Stroke Scale; IV, intravenous thrombolysis; IA, intra-arterial thrombectomy.

The clinical characteristics of the participants are summarized in Table 1 according to clinical outcomes. Before PSM was performed, the patients with unfavorable outcomes significantly differed from those with favorable outcomes in terms of age, sex, hypertension, diabetes mellitus, atrial fibrillation, reduced kidney function, hyperhomocysteinemia, leukocytosis, anemia, stroke subtype, pre-stroke antithrombotic use, poor initial NIHSS, and recanalization therapy. After PSM was conducted, most variables were successfully balanced in patients with favorable and unfavorable outcomes. Patients with favorable and unfavorable outcomes (620 in each category) were matched based on similarities in their demographic and clinical characteristics so that the standardized mean differences between most background factors were within a cut-off value of 0.1 (Figure 2).

The homocysteinemia level was significantly associated with unfavorable outcomes in patients with stroke (Table 2). Not taking pre-stroke antithrombotic medication caused unfavorable outcomes, while taking antithrombotic medication showed a correlation with positive outcomes (Table 2). Specifically, not receiving aspirin (aOR 1.45), APT (aOR 1.57), OAC (aOR 1.33), or either APT or OAC (aOR 1.56) was independently linked with unfavorable clinical outcomes. This relationship remained consistent before and after PSM.

**Figure 3:**
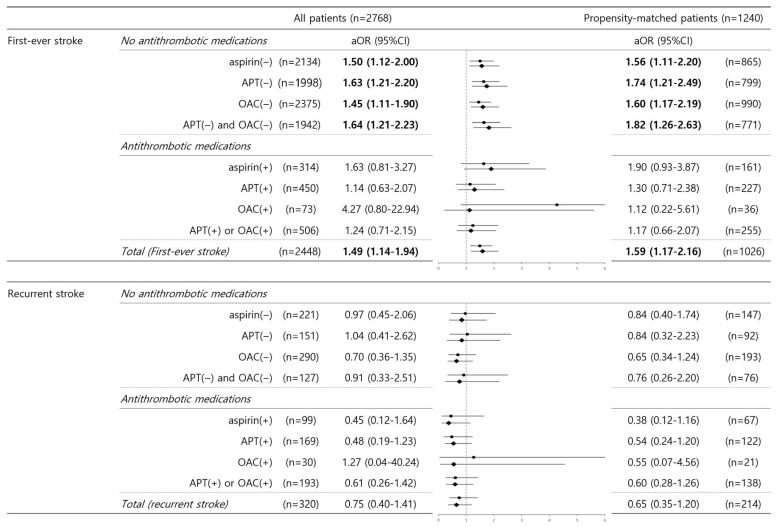
Hyperhomocysteinemia and unfavorable outcomes in terms of stroke recurrence and the use of various antithrombotic medications. In the binary logistic regression analysis of unfavorable outcomes, the adjusted odds ratio of hyperhomocysteinemia was evaluated after stratification for stroke recurrence (first-ever stroke vs. recurrent stroke) and the use of various antithrombotic medications. Data are expressed as adjusted ORs with 95% CIs in parentheses. Values in bold font indicate statistical significance (*p* < 0.05). Values in regular font show no statistical significance. Abbreviations: aOR, adjusted odds ratio; 95% CI, 95% confidence interval; APT, antiplatelet; OAC, oral anticoagulant.

After stroke recurrence was stratified, the relationship between no antithrombotic medication pre-stroke and unfavorable outcomes remained significant in patients who underwent their first-ever stroke (no aspirin, aOR 1.50–1.56; no APT, aOR 1.63–1.74; no OAC, aOR 1.45–1.60; no APT and no OAC, aOR 1.64–1.82) before and after PSM. Among the patients using pre-stroke antithrombotic medications, hyperhomocysteinemia was not related to unfavorable clinical outcomes regardless of first-ever or recurrent stroke events (Figure 3). 

## 4. Discussion

This study investigated the effects of pre-stroke antithrombotic medication and hyperhomocysteinemia on the clinical outcomes of 2768 patients with stroke. The results showed that patients with unfavorable outcomes 3 months after stroke onset exhibited certain clinical characteristics, including hyperhomocysteinemia, antithrombotic medications, and stroke recurrence. After stratification by pre-stroke antithrombotic medication status, the association between hyperhomocysteinemia and unfavorable outcomes was maintained only among patients who did not receive pre-stroke antithrombotic medication. When patients were classified according to stroke recurrence, the relationship between not taking pre-stroke antithrombotic medication and unfavorable outcomes remained significant only in patients experiencing their first-ever stroke but not in those undergoing recurrent stroke. These relationships persisted both before and after PSM. This finding suggests that antithrombotic medications potentially elicit modifying effects on hyperhomocysteinemia in relation to the clinical outcomes of patients with first-ever stroke.

Previous studies on clinical outcomes following stroke reported that the association between hyperhomocysteinemia and unfavorable prognosis is likely mediated by endothelial dysfunction, thrombosis, oxidative stress, enzyme functional modifications, and platelet activation, which collectively contribute to impaired vascular health or subsequent neuropathology [9,10,11,12]. Consistent with the present research, previous studies account for hyperhomocysteinemia and control confounding factors; therefore, hyperhomocysteinemia is significantly associated with unfavorable outcomes 3 months after stroke onset.

Several studies on pre-stroke medications have investigated direct homocysteine-lowering interventions through vitamin B supplementation [13,14,15,16,17,18]. Although some studies have reported conflicting results [17,18], most meta-analyses have concluded that homocysteine-lowering interventions do not significantly reduce the risk of stroke [13,14,15,16]. Another potential intervention for hyperhomocysteinemia involves antithrombotic medications, considering that aspirin or other antiplatelet agents are commonly prescribed; approximately 11% to 54% of adults regularly take these medications to avoid cardiovascular and cerebrovascular diseases [31]. Previous studies also revealed conflicting findings on the relationship between antithrombotic medications and hyperhomocysteinemia [18,19,20,21,22,23]. These inconsistencies include reports that homocysteine-lowering therapy can prevent stroke in patients not receiving antiplatelets [18,19,21]; the risk of recurrent stroke associated with high-dose vitamin B therapy increases among patients concurrently using antiplatelets [20]; and certain antiplatelet agents or their combinations reduce stroke recurrence specifically among patients with hyperhomocysteinemia [22,23]. However, these studies have several limitations; for instance, they evaluated the risk of stroke, such as recurrence, rather than clinical outcomes [18,19,20]. Furthermore, they measured only short-term outcomes upon hospital discharge or within 7 days post-stroke without considering pre-stroke antithrombotic medication status [21]. Other studies exclusively assessed antiplatelet agents without evaluating anticoagulants, although both medication classes are commonly used in secondary stroke prevention [18,19,20,21,22]. Some studies also relied primarily on post hoc analyses [19,20,22,23]. A previous study investigating oral anticoagulation therapy in the context of hyperhomocysteinemia reported an increased risk of cardiovascular events, but not specifically strokes, among patients with hyperhomocysteinemia receiving oral anticoagulants such as warfarin [32]; however, it did not evaluate clinical stroke outcomes. Conversely, the present study demonstrates that antiplatelet and anticoagulant therapies significantly influence clinical outcomes after stroke in patients with hyperhomocysteinemia.

The present study shows that the association between pre-stroke antithrombotic medications and clinical outcomes varies according to stroke recurrence status, specifically between first-ever and recurrent strokes. Recurrent stroke is related to certain risk factors, including stroke subtypes such as LAD or CE, hypertension, diabetes mellitus, and high stroke severity [33]. Factors such as renal function influence the association between homocysteine levels and recurring stroke [34]. We hypothesized that these variations in underlying conditions or clinical characteristics between first-ever and recurrent strokes may explain the differential effects of pre-stroke antithrombotic medication on clinical outcomes. Specifically, in patients who experienced their first-ever stroke but were not receiving pre-stroke antithrombotic medication, hyperhomocysteinemia was significantly associated with unfavorable outcomes. Conversely, in patients with recurrent stroke, hyperhomocysteinemia was not significantly related to unfavorable outcomes, irrespective of pre-stroke antithrombotic medication use. However, given the retrospective nature of our study, definitive conclusions cannot be drawn, and further prospective research should be conducted to confirm these observations.

This study has the following limitations: First, it is a retrospective study. As such, although not taking pre-stroke antithrombotic medication was closely related to unfavorable outcomes in patients with hyperhomocysteinemia and first-ever stroke, this finding implies only an association and not a causal relationship. Thus, the causal relationship should be verified through additional prospective studies. Second, this single-center observational study included a single ethnic population; consequently, inevitable selection bias may have occurred. Therefore, our results might not be representative of the general population. Third, information on vitamin therapy or other conditions related to homocysteine, such as hypothyroidism, lifestyle, and genetic factors and folate, vitamin B_6_, or vitamin B_12_ supplements, was not obtained and could have contributed to the interpretation of results [3,35]. Fourth, studies have reported different effects of various antiplatelet combinations on homocysteine levels and the risk of stroke [22,23]. Fifth, clinical outcomes after stroke onset should account for the contribution of rehabilitation interventions, such as structured neurophysiological programs or robotic-assisted rehabilitation [36,37,38]. Further studies should investigate the clinical outcomes of stroke in relation to homocysteine levels by considering specific combinations of various medications and rehabilitation programs undertaken by patients.

## 5. Conclusions

This study correlates unfavorable outcomes with the non-use of pre-stroke antithrombotic medication and hyperhomocysteinemia in patients who have experienced their first-ever stroke. Therefore, pre-stroke antithrombotic medications could be associated with homocysteine levels and clinical outcomes in patients with specific stroke types. Elucidating the role these factors play during stroke could improve our understanding and management of patients experiencing stroke.

## Figures and Tables

**Table 1:** Demographic and clinical characteristics according to clinical outcomes.

Variables	All Patients (*n* = 2768)			Propensity-Matched Patients (*n* = 1240)		
	Favorable Outcome (*n* = 1920)	Unfavorable Outcome (*n* = 848)	*p*	Favorable Outcome (*n* = 620)	Unfavorable Outcome (*n* = 620)	*p*
Age, ≥65 years	836 (43.52%)	611 (72.1%)	<0.001	389 (62.7%)	407 (65.6%)	0.314
Sex, men	1277 (66.5%)	462 (54.5%)	<0.001	364 (58.7%)	348 (56.1%)	0.358
Hypertension	1173 (61.1%)	612 (72.2%)	<0.001	451 (72.7%)	435 (70.2%)	0.346
Diabetes mellitus	630 (32.8%)	312 (36.8%)	0.045	232 (37.4%)	235 (37.9%)	0.907
Dyslipidemia	981 (51.1%)	414 (48.8%)	0.284	307 (49.5%)	311 (50.2%)	0.865
Recurrent stroke	157 (8.2%)	163 (19.2%)	<0.001	105 (16.9%)	109 (17.6%)	0.822
Atrial fibrillation	269 (14.0%)	235 (27.7%)	<0.001	113 (18.2%)	142 (22.9%)	0.049
Reduced kidney function	206 (10.7%)	218 (25.7%)	<0.001	121 (19.5%)	147 (23.7%)	0.084
Hyperhomocysteinemia	315 (16.4%)	220 (25.9%)	<0.001	120 (19.4%)	151 (24.4%)	0.039
Leukocytosis	147 (7.7%)	109 (12.9%)	<0.001	65 (10.5%)	81 (13.1%)	0.186
Anemia	292 (15.2%)	231 (27.2%)	<0.001	148 (23.9%)	157 (25.3%)	0.598
Subtype of stroke (TOAST)			<0.001			0.871
LAD	390 (20.3%)	218 (25.7%)	(<0.001)	152 (24.5%)	155 (25.0%)	(0.900)
CE	290 (15.1%)	195 (23.0%)	(<0.001)	112 (24.5%)	122 (19.7%)	(0.510)
SVO	697 (36.3%)	187 (22.1%)	(<0.001)	173 (27.9%)	168 (27.1%)	(0.800)
OD+UD	543 (28.3%)	248 (29.2%)	0.620	183 (29.5%)	175 (28.2%)	(0.660)
Antithrombotics use						
Aspirin	253 (13.2%)	160 (18.9%)	<0.001	121 (19.5%)	107 (17.3%)	0.341
Any APT	382 (19.9%)	237 (27.9%)	<0.001	185 (29.8%)	164 (26.5%)	0.207
Any OAC	45 (43.7%)	58 (56.3%)	<0.001	17 (2.7%)	40 (6.5%)	0.003
APT or OAC	412 (21.5%)	287 (33.8%)	<0.001	196 (31.6%)	197 (31.8%)	1.000
Poor initial NIHSS	229 (11.9%)	466 (55.0%)	<0.001	219 (35.3%)	238 (38.4%)	0.289
Recanalization therapy						
Intravenous	142 (7.4%)	120 (14.2%)	<0.001	83 (13.4%)	89 (14.4%)	0.681
Intra-arterial	32 (1.7%)	53 (6.2%)	<0.001	25 (4.0%)	30 (4.8%)	0.582

Data are expressed as the raw count (percentages). *p* values in parentheses are indicated post hoc after the chi-square test. Abbreviations: TOAST, Trial of Org 10172 in Acute Stroke Treatment; LAD, large artery disease; CE, cardioembolism; SVO, small vessel occlusion; OD, stroke of other determined etiology; UD, stroke of undetermined etiology; APT, antiplatelet; OAC, oral anticoagulant; NIHSS, National Institutes of Health Stroke Scale.

**Table 2:** Hyperhomocysteinemia and clinical outcome of patients with stroke based on their use of various antithrombotic medications.

Use of Various Antithrombotic Medications		Crude OR (95% CI)	Adjusted OR (95% CI)
*No antithrombotics*			
aspirin(−)	(*n* = 2355)	**1.93 (1.56**–**2.40)**	**1.45 (1.11**–**1.89)**
APT(−)	(*n* = 2149)	**2.09 (1.66**–**2.62)**	**1.57 (1.19**–**2.08)**
OAC(−)	(*n* = 2665)	**1.74 (1.43**–**2.14)**	**1.33 (1.04**–**1.71)**
APT(−) and OAC−)	(*n* = 2069)	**2.05 (1.62**–**2.61)**	**1.56 (1.17**–**2.09)**
*Antithrombotic medicines*			
aspirin(+)	(*n* = 413)	1.15 (0.73–1.81)	1.08 (0.61–1.91)
APT(+)	(*n* = 619)	1.08 (0.74–1.57)	0.94 (0.59–1.52)
OAC(+)	(*n* = 103)	1.84 (0.76–4.47)	2.68 (0.85–8.47)
APT(+) or OAC(+)	(*n* = 699)	1.15 (0.82–1.63)	1.02 (0.66–1.59)
*Total (all stroke)*	(*n* = 2768)	**1.78 (1.47**–**2.17)**	**1.36 (1.07**–**1.73)**

Values in bold font indicate statistical significance (*p* < 0.05). Abbreviations: OR, odds ratio; 95% CI, 95% confidence interval; APT, antiplatelet; OAC, oral anticoagulant.

## Data Availability

The raw data supporting the conclusions of this article will be made available by the authors upon request.
